# The emerging role and therapeutic implications of bacterial and parasitic deubiquitinating enzymes

**DOI:** 10.3389/fimmu.2023.1303072

**Published:** 2023-11-22

**Authors:** Markus Wehrmann, David Vilchez

**Affiliations:** ^1^ Cologne Excellence Cluster for Cellular Stress Responses in Aging-Associated Diseases (CECAD), University of Cologne, Cologne, Germany; ^2^ Institute for Integrated Stress Response Signaling, Faculty of Medicine, University Hospital Cologne, Cologne, Germany; ^3^ Center for Molecular Medicine Cologne (CMMC), University of Cologne, Cologne, Germany; ^4^ Institute for Genetics, University of Cologne, Cologne, Germany

**Keywords:** ubiquitin, deubiquitinating enzymes (DUBs), infection mechanisms, immune responses, autophagy and apoptosis

## Abstract

Deubiquitinating enzymes (DUBs) are emerging as key factors for the infection of human cells by pathogens such as bacteria and parasites. In this review, we discuss the most recent studies on the role of deubiquitinase activity in exploiting and manipulating ubiquitin (Ub)-dependent host processes during infection. The studies discussed here highlight the importance of DUB host-pathogen research and underscore the therapeutic potential of inhibiting pathogen-specific DUB activity to prevent infectious diseases.

## Introduction

Ubiquitination is a pivotal cellular process that mediates protein turnover through the ubiquitin proteasome system (UPS) (1). Moreover, the precise regulation of ubiquitination is essential for proper cellular function, as it controls protein localization, activates DNA repair pathways, and enables protein function and protein-protein interactions (2). Ubiquitination is achieved through a sequential mechanism involving E1, E2 and E3 enzymes. This enzymatic cascade forms an isopeptide bond between the epsilon-amino group of a lysine (K) on the target protein and the carboxyl-group of the last residue (G76) in the Ub moiety. The ubiquitination cascade can also link additional Ub molecules to the internal lysine sites of the first Ub, forming a Ub-chain (3, 4). In recent years, the role of unique Ub-signals, such as M1-linked linear ubiquitination, has been increasingly recognized. In contrast to typical Ub-signaling chains, these chains are assembled via the N-terminal methionine by the linear Ub chain assembly complex (LUBAC) (5). M1-linked linear ubiquitination plays a pivotal role in regulating NF-κB activity, cell death, inflammation and immunity against infectious pathogens. Consequently, genetic dysfunction in this pathway can increase susceptibility to infectious diseases (5, 6).

In addition to Ub-ligases, the stringent control of ubiquitination levels relies on the activity of DUBs, which are subsequently crucial for maintaining cellular homeostasis, function and viability (7). DUBs are proteases that hydrolyze peptide or isopeptide bonds between Ub molecules in Ub-chains. They can also catalyze the cleavage of isopeptide bonds between Ub and a modified protein (4, 8, 9). Thus, DUBs have a critical role in cleaving polyUb-chains and removing Ub moieties from proteins once they have fulfilled their designated functions. Consequently, these enzymes also contribute to maintaining a constant pool of intracellular free Ub molecules that can be reused in following signaling events. Eukaryotic DUBs can be classified into seven different subclasses, six of which are cysteine proteases (UCH, USP, OTU, Josephin, MINDY and ZUFSP), while the seventh belongs to the metalloprotease family (JAMM) (10). Each subtype is defined based on their structural and sequence homology, as well as its preference and affinity towards different linkage types of Ub chains and protein substrates, which are discussed in more detail in other reviews (10–13).

Given the direct role that DUBs have in regulating cellular function and homeostasis, it is not surprising that pathogens utilize DUB function to exploit Ub-dependent host pathways to progress infection. Although it has long been known that pathogenic microbes and viruses manipulate the Ub system during infection, the primary focus until recently was on bacterial effectors that mimic and hijack Ub-E3 ligases (14–17). However, a wide range of DUB-dependent mechanisms are now emerging as key targets for exploitation during infection by bacteria and parasites. Interestingly, bacteria do not use the UPS for cellular regulation or encode Ub in their genome (10). However, several species express bacteria-exclusive proteases of the CE-Clan family that exhibit DUB activity towards K63-linked Ub chains and Ub-like proteins (18). The latter suggests an important function of DUBs in the infection process of eukaryotic cells (10). In this review, we summarize and compare different DUB mechanisms used by parasites and bacteria throughout the different steps of the infectious cycle. Furthermore, we highlight the most promising areas of DUB research in terms of future directions and therapeutic potential.

## Maintenance of the replication niche and hijacking of host trafficking pathways

**Figure 1:**
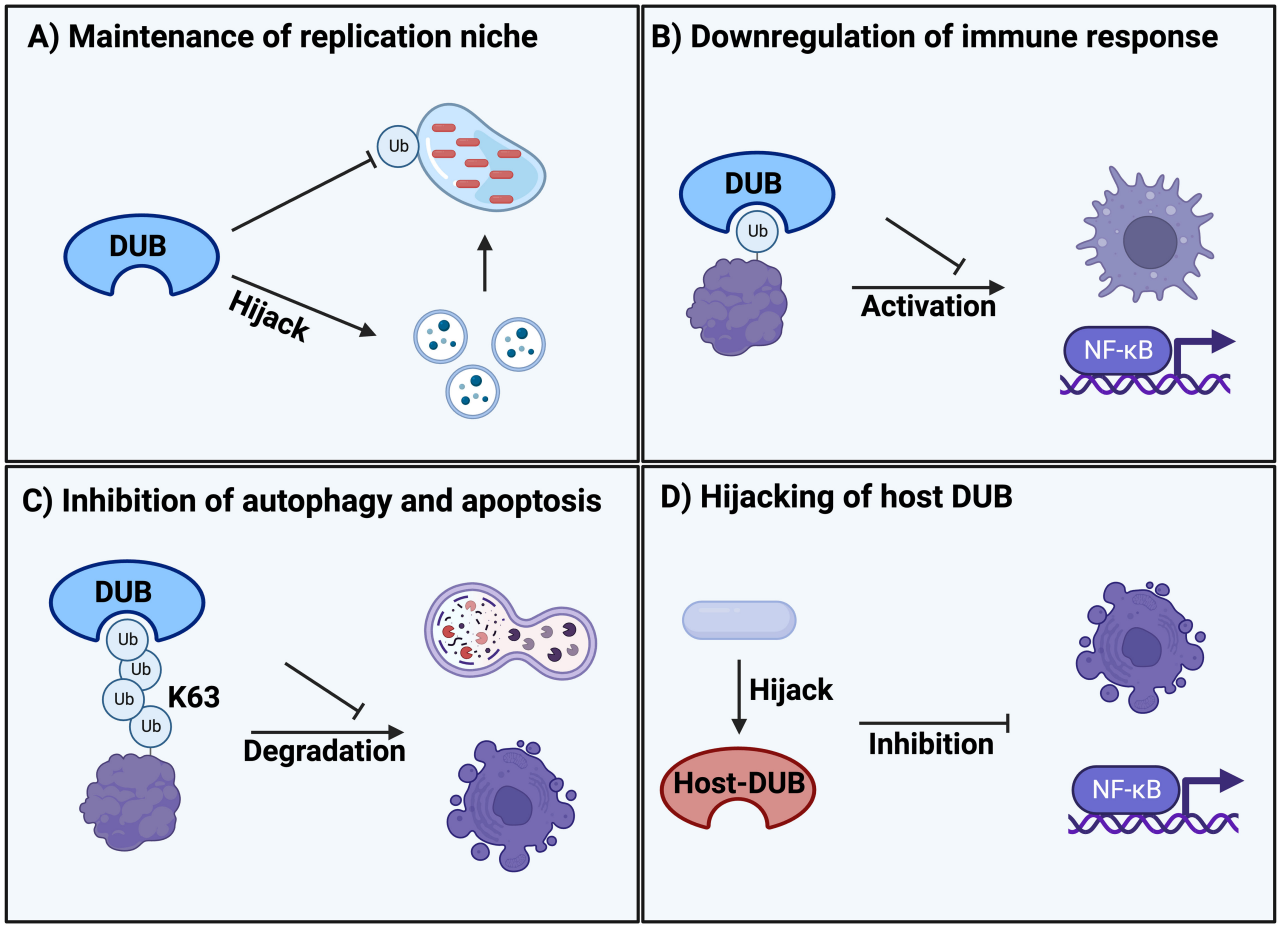
Classification of different DUB mechanisms during bacterial and parasitic infections. **(A)** Maintenance of replication niche inside the host by clearance of ubiquitination on the vacuole membrane and hijacking of trafficking pathways. **(B)** Downregulation of the immune system response such as macrophages and NF-κB signalling due to infectious DUB activity. **(C)** Protection from degradation by removing K63-polyubiquitination signals that are responsible for regulating autophagy and apoptosis. **(D)** Hijacking and manipulation of host DUBs upon entrance and infection of the cell that are regulating cellular defence systems such as apoptosis and NF-κB signalling.

An evolutionary conserved strategy for bacteria and pathogens to survive inside the hostile host cell environment during infection is the formation of replication niches, such as bacteria-containing vacuoles (BCVs). To create these vacuoles, bacteria hijack the vesicles secreted by the endoplasmic reticulum (ER) and Golgi as well as downstream trafficking pathways, redirecting them to the infection site for fusion with the bacterial phagosome. This process results in the formation of a membrane-bound vacuole that serves as a replication site (19). This infection strategy is used by numerous bacteria, including *Legionella pneumophila, Coxiella burnetii*, and representative members of the *Salmonella* family. The replication niche inside vacuoles is critical for pathogen survival and virulence because it provides a protective layer and a mechanism to interact with and hijack host cell organelles. This interaction is often mediated and dependent on pathogenic DUB activity (20, 21) (**Figure 1A**, **Table 1**).

**Table 1:** Reported DUB enzymes important in infectious diseases.

Protein	Organism	Function	Ub-chain	Reference
TssM	*B. pseudomallei*	TNFR-associated factor-3 and lipopolysaccharide deubiquitination	K48/63	(22, 23)
EmcB	*C. burnetii*	blocks RIG-I mediated type I IFN production	K63	(24)
ChlaOTU	*C. caviae*	NDP52 binding	K48/63	(25)
ChlaDUB1	*C. trachomatis*	Golgi fragmentation	K63	(26)
ChlaDUB2	*C. trachomatis*	Golgi fragmentation	K63	(26)
Cdu1	*C. trachomatis*	Deubiquitination of apoptosis regulator Mcl-1	K48/63	(25)
ElaD	*E. coli*	Macrophage killing?	K63	(27)
USP48	*H. pylori*	Reduced apoptotic cell death and NF-κB immune signalling by RelA deubiquitination	K48	(28)
A20	*L. donovani*	Silencing of TLR2-mediated proinflammatory response	K63	(29)
RavZ	*L. longbeachae*	Deconjugation of LC3 autophagy related protein	unknown	(30–32)
DupA	*L. pneumophila*	Vacuole maintenance	PR-Ub.	(33, 34)
DupB	*L. pneumophila*	Vacuole maintenance	PR-Ub	(33, 34)
LotA	*L. pneumophila*	Vacuole maintenance	K6	(35)
LotB	*L. pneumophila*	SNARE complex manipulation	K63	(36)
LotC	*L. pneumophila*	Rab10 deubiquitination	K6, K11, K48	(37)
MavC	*L. pneumophila*	Deubiquitination of UBE2N	K63	(38)
MavcA	*L. pneumophila*	Inhibition of NF-κB immune signalling	K63	(38)
RavD	*L. pneumophila*	Clearance of M1-linked linear Ub chains	M1 Ub	(39)
SidJ	*L. pneumophila*	Deubiquitination of Rab33b, regulation of SidE effectors	K63-Di-Ub	(40)
SdeA/B/C	*L. pneumophila*	Broad base deubiquitination	K63	(41)
OtDUB	*O. tsutsugamushi*	Inactivation of T-cells and the innate immune response?	K33-Di-Ub	(42)
SpvD	*S. Typhimurium*	Inhibits nuclear translocation of p65	Unknown	(43, 44)
SseL	*S. Typhimurium*	Macrophage toxicity	K63	(45)
YopJ	*Y. pestis*	STING deubiquitination and inhibition transport of IRF3 signalling	K63	(46)

In *Legionella pneumophila*, a gram-negative bacterium that causes Legionnaires’ disease, Lot class DUBs are localized in the vacuolar membrane to establish the replication vacuole during infection. Several well-characterized members of the Lot DUB class (LotA, LotB and LotC) are essential for infection. For instance, LotA localizes to the vacuolar membrane and displays dual catalytic activity specific to two different types of Ub chains by harboring two Ub-binding domains (35). However, unlike other DUBs, one of these domains uniquely shows high specificity for K6-linked polyUb chains. K6-linked polyubiquitination has been associated to parkin-mediated autophagic degradation of mitochondria and intracellular bacteria (37, 47). It will be exciting to understand this so far unexplored mechanism during infection and its potential connection with mitophagy-related processes.

In contrast to LotA, LotB exhibits a highly specialized role in manipulating v-SNARE complexes of the early secretory pathway. LotB activity reverses K63-linked ubiquitination of Sec22b, facilitating dissociation of the t-SNARE syntaxin-3 from Sec22b, which subsequently attaches to the *Legionella*-containing vacuole (LCV) (36). Similarly, LotC shows fine-tuned control over Rab10 ubiquitination in combination with the two bacterial E3-Ub-ligases SidC and SdcA (37). Rab10 is a GTPase involved in membrane trafficking, which is required for maximizing replication potential as well as generating and maintaining the LCV of *L. pneumophila* (48). Generally, the ability of both attaching and removing Ub from the same substrates in Golgi trafficking pathways highlights the precise control that bacterial effectors exert over cellular pathways to establish the replication vacuole.

Another set of DUB enzymes identified in *L. pneumophila* are DupA and DupB. Generally, effectors of the SidE family of *L. pneumophila* are responsible for unconventional conjugation of Ub to serine residues of host proteins via phosphoribosyl linker (PR-ubiquitination) (33). During PR-ubiquitination, instead of the canonical ubiquitination cascade involving E1, E2 and E3 enzymes, SidE effectors harbor different functional domains and can perform the entire process of attaching Ub to serine or tyrosine residues using NAD+ instead of the conventional ATP (49, 50). This process is suggested to be important for establishing the replicative niche for intracellular growth (51). Recently, it was reported that the bacterial DUBs DupA and DupB are important in the regulation of host protein PR-ubiquitination by bacterial effectors (33). Proteomics analysis of *ΔdupA* and *ΔdupB Legionella* mutants revealed that many PR-deubiquitination substrates of DupA and DupB were recruited to the LCV and are involved in vesicle transport and trafficking between the ER and Golgi apparatus. This indicates an important but not yet fully understood role of PR-ubiquitination signaling in hijacking host trafficking pathways and maintaining bacterial vacuoles. Such mechanisms could potentially be manipulated to prevent infection (34).

A less studied pathogen is *Chlamydia trachomatis*. Nevertheless, the two DUB orthologs ChlaDUB1 and ChlaDUB2 are important for the regulation of its virulence and pathogenesis. Both ChlaDUB1 and 2 have specificity for K63-linked Ub-chains, being necessary and sufficient to induce fragmentation of the Golgi apparatus to hijack trafficking pathways. This highlights their importance in establishing the replicative niche during pathogenesis (26).

Collectively, bacteria utilize DUBs with highly specialized and diversified roles with different Ub-chain specificities that reside on the vacuolar membrane to hijack Golgi trafficking pathways. As such, these DUBs enable replication and survival in a protected niche inside the host cell. Thus, intervening with DUB activity that is indispensable for establishment of the replication niche and bacterial survival could open up a therapeutic avenue to prevent and treat infection.

## Downregulation of immune response

The most prevalent and reported strategy of bacterial and parasitic DUB activity is evasion and downregulation of the immune response. The primary transcription factor NF-kB is a key regulator of the host immune defense system upon infection (52). Thus, NF-kB is a prime target for downregulation through deubiquitination of activating factors (**Figure 1B**, **Table 1**).

Recent studies have described how DUBs mediate the downregulation of NF-kB signaling within macrophages during infection. In studies focused on *L. pneumophila*, several DUBs were found to act on different host substrates during the infectious cycle of the bacterium. In particular, the two effector orthologs, MavC and MvcA, possess the ability of both non-canonical ubiquitination and deubiquitination of their target substrates. MavC catalyzes ubiquitination of the host E2 conjugating enzyme UBE2N, inhibiting the formation of K63-linked Ub-chains and thus impeding activation of NF-kB signaling (38).

Another example is the DUB RavD, one of the first bacterial DUBs identified capable of cleaving linear Ub-chains (39). RavD DUB activity prevents accumulation of M1-linked linear Ub-chains on *Legionella* vacuoles inside the host cell. These chains have an important regulatory role in NF-kB activation and subsequent inflammation. Indeed, its dysregulation has been associated with several human pathologies (6). Similarly, the cysteine hydrolase SpvD expressed by *S. Typhimurium*, which exhibits a DUB-like structural fold, is responsible for inactivation of an NF-kB promoter, thereby inhibiting the proinflammatory response by preventing nuclear translocation of the transcription factor p65 (43, 44).

In other bacterial species, such as the Salmonella family, the well-studied effector protein SseL from *Salmonella enterica* serovar *Typhimurium* was shown to possess hydrolase and DUB activity towards K63-linked Ub-chains. While SseL function is not essential for bacterial replication, it is indispensable for virulence and cytotoxicity in macrophages. This indicates that deubiquitination of SseL substrates contribute to macrophage killing, but not inhibition of NF-kB signaling (45), and is thus an alternate means by which the host immune response can be compromised through DUB activity. An ortholog of SseL, named ElaD, with high Ub-binding affinity and deubiquitination activity, has also been identified in pathogenic *E. coli*. However, its effect on virulence remains to be assessed (27).

Evading the immune response is also adopted by other bacterial species like *Yersinia pestis*. The DUB YopJ is responsible for K63-linked deubiquitination of STING, thereby preventing complex formation with TBK1 and inhibiting multiple immune activators (IRF3 and NF-κB signaling) (46). Similarly, a recently discovered DUB from *Orientia tsutsugamushi* (OtDUB) has strong activity towards K33-linked diUb and polyUb chains of various kinds (42). K33-linked diUb plays a role in activation of T-cells and the innate immune response in general (53). In this way, *O. tsutsugamushi* may downregulate general immune responses upon infection by abolishing Ub-signals.

The effector DUB EmcB expressed by *Coxiella burnetii* employs a different strategy, inhibiting detection by the immune system rather than reducing downstream responses like NF-kB signaling. Usually, type I interferon (IFN) production is induced during infection when nucleic acids are released into the host cytosol, leading to the interaction of RIG- I with the adaptor protein MAVS (54). However, EmcB cleaves the K63-specific activating signal of RIG- I, thus preventing type I interferon production during *C. burnetii* infection. This is an elegant way to evade the immune system at the initial step, allowing the pathogen to persist inside host cells (24).

Finally, the *B. pseudomallei* DUB TssM was reported to suppress both NF-kB signaling and type I IFN pathway by cleaving both K48- and K63-linked Ub-chains. Subsequently, knockout of this DUB led to increased inflammation. Furthermore, increased TssM was found in human samples after bacterial infection (22). Moreover, a recent study revealed a bacterial esterase function of TssM, which is structurally independent of its isopeptidase activity (23). The latter activity is responsible for directly reversing the lipopolysaccharide ubiquitination of RNF213, an important regulator in restricting bacterial growth by attaching polyUb and autophagic receptors to the bacterial replication site (23, 55). Consequently, the esterase activity of TssM is an important regulator of *B. pseudomallei* virulence, as it hinders its detection by the immune system and inhibits its autophagic degradation.

Together, these studies demonstrate that bacterial DUBs have evolved specialized roles in downregulating and evading the immune response in distinct ways that are crucial for the infection process. Furthermore, the ability of pathogens to coordinate both ubiquitination and deubiquitination of host proteins by acting in concert with E3 ligases is complex and tightly regulated. Therefore, potential therapeutic strategies must consider a multifaceted approach.

## Protection from degradation and apoptosis

Autophagy plays a key role in the direct removal of microorganisms via degradation, control of inflammation and activation of the immune system, making it an important host defense mechanism during infection (56). Similarly, apoptotic cell death is a common response to infection, triggered and inhibited by various pathogenic effectors (57). Thus, it is a common strategy for pathogens to inhibit autophagic degradation and apoptotic cell death during infection and replication (**Figure 1C**, **Table 1**).

The effector RavZ was identified as a functional and important DUB in various *Legionella* species and was one of the first examples of bacterial effector proteins that manipulate host autophagy (30–32). RavZ harbors cysteine protease activity and hydrolyzes lipid-conjugated LC3, a Ub-like protein incorporated in autophagic processes on autophagosome membranes (58). This hydrolysis reduces the interaction of LC3 with other autophagosome proteins, thereby downregulating autophagy (30). Moreover, further studies revealed the interference of Ub recruitment to the replication vacuoles during *Legionella* infection upon RavZ activity (31, 32). This raises the question of whether RavZ harbors DUB-like activity of RavZ in addition to its general cysteine protease activity towards lipid-conjugated substrates. Future studies may shed light on RavZ’s dual activity towards both lipid conjugated substrates and Ub. This could describe a highly sophisticated system of manipulating several host pathways using, potentially opening the door to therapies for *Legionella* infection.

Moreover, it was reported that the *Legionella* DUB SidJ regulates the bacterial effector family SidE, which are important for the dynamics of phagosome ubiquitination during infection (41). The SidE family consists of different bacterial effectors such as SidE, SdeA, SdeB and SdeC that mediate both ubiquitination and removal of PR-ubiquitination (41, 49). Structural analysis of these enzymes revealed unique Ub-binding and contact domains that allow for deubiquitination of three different Ub-chain types with preference for K63 Ub-chains (41). In particular, SidJ was shown to remove ubiquitination from the host protein Rab33b in infected cells (59). Rab33b is involved in autophagy as a modulator of autophagosome formation. Thus, it is possible that DUB activity of SidJ inhibits autophagy and pathogenic degradation during infection (60). Additionally, SidJ activity suppresses the toxicity of SidE effectors by removing them from the bacterial phagosome (40).

Autophagy-inhibiting DUBs are also found in the *Chlamydiae* phylum. The *Chlamydia caviae* effector *Chla*OTU, while dispensable for infectious activity, alleviates detrimental Ub accumulation at the pathogen entry site. *Chla*OTU is capable of binding both Ub and NDP52 with distinct protein domains. NDP52 is an autophagic receptor protein involved in selective autophagy of microorganism during infection (61). Thus, *Chla*OTU may inhibit autophagic degradation through binding and modulating NDP52.

In contrast, the vacuolar membrane-bound DUB Cdu1 expressed by *Chlamydia trachomatis* was shown to interact with the apoptosis regulator Mcl-1. In particular, deubiquitination of Mcl-1 by Cdu1 leads to its stabilization and prevents proteasomal degradation, thus inhibiting cellular apoptosis in favor of bacteria replication (25).

These examples highlight the multiple layers of control that pathogenic DUB activity enables over the host Ub-pathway through interaction and inhibition of host proteins involved in autophagy and apoptosis during infection. Thus, this complexity needs to be considered when designing new therapeutic agents.

## Hijacking of host DUB enzymes

The ubiquitination system is responsible for regulating many different cellular and physiological processes. Here, host DUB activity also contributes to the regulation of processes such as IFN-I signaling and host defense activities in general (62). This offers a potential hijacking mechanism that bacteria can exploit for infection (**Figure 1D**, **Table 1**).

There have been only a few reported cases in which host cell DUBs were manipulated by bacteria and parasites upon infection. The parasite *Leishmania donovani* is capable of replicating within macrophages by downregulation of TLR-mediated inflammatory responses of the host cell. Upon *L. donovani* infection, the ubiquitination of TRAF6 is reduced, which subsequently inhibits the assembly of the TRAF6–TAK1–TAB2 complex (29). This complex is an efficient activator of NF-kB signaling and mediator of immune response in general (63). Further investigations of this mechanism revealed that *L. donovani* infection induces the host DUB A20, which is responsible for the increased deubiquitination of TRAF6 and thus inhibition of TLR response upon infection (29). This is an interesting example of pathogens exploiting the innate negative feedback systems of host cells to decrease the immune response. It would thus be interesting to assess whether infection can be counteracted by blocking this negative feedback loop exploited by pathogens.

A similar mechanism involving the exploitation of the host DUB A20 is found during the infection with the human pathogen *Helicobacter pylori* (28, 64). Typically, NF-kB activity is regulated by DNA binding of the p50 and RelA heterodimer (52). Termination of the activity is regulated by K48-ubiquitination dependent proteasomal degradation of RelA (65). Thus, during infection with *H. pylori*, the host DUB USP48 stabilizes RelA by removing the K48 degradation signal which in turn increases the transcription of the host DUB A20. Subsequently, increased A20 deubiquitinating activity leads to the inhibition of caspase-8-dependent apoptotic cell death and suppression of NF-κB activation, prolonging survival and colonization of the pathogen (28, 64).

Therefore, the inhibition of apoptotic cell death and immune signaling by hijacking of host DUBs enables successful colonization of macrophages and infection of human cells. It will be interesting to see how our understanding of exploiting host negative feedback loops for inactivation of defense mechanisms, such as apoptotic cell death and NF-kB activity, will be expanded in the future and if this could potentially be exploited to design specific therapeutic strategies.

## Discussion

The manipulation of Ub-dependent host pathways through DUB activity is emerging as a key mechanism employed by bacteria and parasites during infection. It is fascinating to observe the highly sophisticated systems revolving around DUBs that enable successful infection. First, a large variety of biochemical mechanisms regulating preference to distinct Ub-chains as well as bifunctional enzymes are utilized by the pathogens. These complex systems illustrate the competitive co-evolution of pathogens and hosts. In addition, distinct pathogens exhibit a tight control over the removal and attachment of Ub, thereby regulating the activation and inactivation of regulatory components of the host cell. This process often involves cooperation with bacterial E3 ligases.

Besides, DUB activity interferes with a variety of Ub-dependent cellular functions and many different host substrate proteins are targets for deubiquitination by different pathogens (**Figure 1**). In addition, most pathogens exploit different mechanisms of DUB activity throughout the infectious cycle. Indeed, the same bacteria can target different substrate proteins to manipulate distinct host pathways and ensure successful infection (**Table 1**). This complexity underscores the challenges that host cells must overcome to prevent infection. Since many parasitic DUBs remain uncharacterized, the focus of this review was mainly on bacterial DUB activity. However, it will be interesting to follow how more examples of parasitic DUBs will be revealed in the future.

Considering the essential role of DUB activity in various infection stages and across different pathogens, the application of DUB inhibitors could present a promising therapeutic strategy for the treatment of infectious diseases (66). For instance, designing DUB inhibitors to specifically inhibit bacterial DUBs could lead to effective therapies for diseases that have eluded targeted treatment so far. Furthermore, the infection-specific interaction between indispensable DUBs and their cellular host substrates can be exploited for structural inhibition of these interactions to prevent infection. In this regard, it will be crucial to consider the complex and tight control that pathogenic DUBs possess over host processes when developing new therapies. The specificity of DUB inhibitors in selectively targeting bacterial DUBs is a crucial factor to consider for therapeutic applications, as inhibiting host DUBs could be detrimental to the cell and potentially exacerbate the infectious process. Interestingly, DUB activity, along with its dysregulation and inhibition is also investigated and proved to be beneficial in the prevention of cancer, neurodegenerative disorders, and aging, which is a risk factor for multiple diseases in general (67–69).

In conclusion, bacterial and parasitic DUB activity during infection can be defined and summarized in different categories that will be further expanded in future research. The importance of these mechanisms highlights the emerging role of DUB activity during infection and opens up the possibility of novel therapeutic to prevent these diseases in the future.

## Author contributions

DV: Writing – review & editing. MW: Writing – original draft.
